# Supplementary material for: Association between bone marrow adipose tissue, abdominal adipose tissue distribution, and volumetric bone mineral density in the Chinese adult population: a retrospective cohort study
Source: PeerJ. 2025 Dec 4;13:e20446. doi: 10.7717/peerj.20446 (PMC12682217; doi:10.7717/peerj.20446)
Supplement: Supplemental Information 2 [file peerj-13-20446-s002.docx]

**Codebook for Categorical Variables**

Sex：0~Female；1~Male；

BMI group: 1~Low BMI; 2~Normal BMI; 3~Overweight; 4~Obesity

Smoking : 0~ No smoking; 1~Smoking

Drinking: 0~ No alcohol drinking; 1~ Alcohol drinking.
