# Supplementary material for: Association between bone marrow adipose tissue, abdominal adipose tissue distribution, and volumetric bone mineral density in the Chinese adult population: a retrospective cohort study
Source: PeerJ. 2025 Dec 4;13:e20446. doi: 10.7717/peerj.20446 (PMC12682217; doi:10.7717/peerj.20446)
Supplement: Supplemental Information 3 [file peerj-13-20446-s003.docx]

STROBE Statement—checklist of items that should be included in reports of observational studies

|  | Item No. | Recommendation | Page  No. | Relevant text from manuscript |
| --- | --- | --- | --- | --- |
| **Title and abstract** | 1 | (*a*) Indicate the study’s design with a commonly used term in the title or the abstract | 2 | 306 Chinese adult living liver transplant donors were enrolled in this cross-sectional study. |
|  |  | (*b*) Provide in the abstract an informative and balanced summary of what was done and what was found | 2 | Our results indicate there is a negative association between BMAT and BMD. And the impact of abdominal adipose tissue distribution on BMD is dual, SAT exhibits a positive effect on BMD, whereas VAT has a detrimental effect.. |
| Introduction | | | |  |
| Background/rationale | 2 | Explain the scientific background and rationale for the investigation being reported | 4 | Although osteoporosis is more common in older women, both men and women start experiencing bone loss at a younger age, which continues throughout their lives[33]. Nevertheless, as far as we know, most research predominantly focuses on female demographic and is performed with elderly participants. The link between abdominal adipose tissue, BMAT and BMD in younger individuals of both gender remains inadequately understood. Furthermore, these interrelationships may vary across populations with different ethnicity, lifestyle, nutritional habits. The Chinese population, in particular, has been understudied in this context. |
| Objectives | 3 | State specific objectives, including any prespecified hypotheses | 4 | Consequently, this study aim to investigate the association between abdominal adipose tissue, BMAT, and lumbar vBMD in a Chinese adult population of both genders. |
| Methods | | | |  |
| Study design | 4 | Present key elements of study design early in the paper | 4 | This retrospective study was approved by the Institutional Review Board and the Ethics Committee of Beijing Friendship Hospital (2024-P2-211-01). The subjects’ information was de-identified prior to analysis. |
| Setting | 5 | Describe the setting, locations, and relevant dates, including periods of recruitment, exposure, follow-up, and data collection | 4 | This study recruited adult living liver transplant donors who underwent transplantation surgery at the liver transplantation center of Beijing Friendship Hospital, Capital Medical University, between October 2019 and March 2024. |
| Participants | 6 | (*a*) *Cohort study*—Give the eligibility criteria, and the sources and methods of selection of participants. Describe methods of follow-up  *Case-control study*—Give the eligibility criteria, and the sources and methods of case ascertainment and control selection. Give the rationale for the choice of cases and controls  *Cross-sectional study*—Give the eligibility criteria, and the sources and methods of selection of participants | 4 | Inclusion criteria were (a) living liver transplant donors who had underwent both abdominal CT and MRI scans within one month before transplantation surgery; (b) age [≥](https://baike.baidu.com/item/%E2%89%A5/0?fromModule=lemma_inlink) 18 years; (c) accessibility of demographic and clinical data. |
|  |  | (*b*) *Cohort study*—For matched studies, give matching criteria and number of exposed and unexposed  *Case-control study*—For matched studies, give matching criteria and the number of controls per case | NA |  |
| Variables | 7 | Clearly define all outcomes, exposures, predictors, potential confounders, and effect modifiers. Give diagnostic criteria, if applicable | 5-6 | The subcutaneous adipose tissue (SAT) refers to the extra-abdominal adipose tissue area, while the visceral adipose tissue (VAT) area refers to the intra-abdominal adipose tissue within the abdominal cavity, bordered by the rectus, external oblique, lumbar quadrate, and psoas muscles. Semi-automatic measurements of the SAT and VAT areas were conducted using the 'Tissue Composition Module' of the QCT PRO software. The total adipose tissue (TAT) area was determined by adding SAT and VAT.  Given the scan range of abdominal CT of subjects, we choose the L1-L2 for vBMD measurement. The average values of L1 and L2 were used to determine the mean vBMD.  To evaluate BMAT, an elliptic ROI was positioned in each vertebra (L1 to L2), with the ROI placed three times per vertebra, and the BMAT values were averaged to determine the mean PDFF of cancellous bone |
| Data sources/ measurement | 8* | For each variable of interest, give sources of data and details of methods of assessment (measurement). Describe comparability of assessment methods if there is more than one group | 5-6 | In the China guideline for diagnosis criteria of osteoporosis with QCT, at least two lumbar vertebrae of the 1st (L1), 2nd (L2) and 3rd (L3) lumbar vertebrae were recommended as the BMD measurement.  All subjects underwent a chemical shift-encoded sequence called Iterative Decomposition of water and fat with Echo asymmetry and Least Square Estimation-iron quantification (IDEAL-IQ) acquisition with a SIGNA Pioneer 3.0 T MRI system (GE Healthcare, Milwaukee, WI, USA). |
| Bias | 9 | Describe any efforts to address potential sources of bias | NA |  |
| Study size | 10 | Explain how the study size was arrived at | 4 | This study recruited adult living liver transplant donors who underwent transplantation surgery at the liver transplantation center of Beijing Friendship Hospital, Capital Medical University, between October 2019 and March 2024. |

Continued on next page

| Quantitative variables | 11 | Explain how quantitative variables were handled in the analyses. If applicable, describe which groupings were chosen and why | 7 | All statistical analyses were performed using SPSS software version 22.0 (SPSS, Chicago, IL, USA) and R (version 4.4.1, R Foundation for Statistical Computing, Vienna, Austria). |
| --- | --- | --- | --- | --- |
| Statistical methods | 12 | (*a*) Describe all statistical methods, including those used to control for confounding | 7 | All statistical analyses were performed using SPSS software version 22.0 (SPSS, Chicago, IL, USA) and R (version 4.4.1, R Foundation for Statistical Computing, Vienna, Austria). |
|  |  | (*b*) Describe any methods used to examine subgroups and interactions | NA |  |
|  |  | (*c*) Explain how missing data were addressed | NA |  |
|  |  | (*d*) *Cohort study*—If applicable, explain how loss to follow-up was addressed  *Case-control study*—If applicable, explain how matching of cases and controls was addressed  *Cross-sectional study*—If applicable, describe analytical methods taking account of sampling strategy | 7 | All statistical analyses were performed using SPSS software version 22.0 (SPSS, Chicago, IL, USA) and R (version 4.4.1, R Foundation for Statistical Computing, Vienna, Austria). |
|  |  | (*e*) Describe any sensitivity analyses |  |  |
| Results | | | | |
| Participants | 13* | (a) Report numbers of individuals at each stage of study—eg numbers potentially eligible, examined for eligibility, confirmed eligible, included in the study, completing follow-up, and analysed | 7 | Demographic characteristics of all subjects are summarized in **Table 1**. A total of 306 subjects, including 156 males (51.0%) and 150 females (49.0%), were eligible for analysis. The mean age was 38.1 ± 9.5 years (range 19-66 years). |
|  |  | (b) Give reasons for non-participation at each stage | 4 | Finally, as presented in **Figure. 1**, there were 306 donors of living liver transplant donors aged over 18years without known medical conditions affecting bone metabolism were included. |
|  |  | (c) Consider use of a flow diagram | 4 | Finally, as presented in **Figure. 1**, there were 306 donors of living liver transplant donors aged over 18years without known medical conditions affecting bone metabolism were included. |
| Descriptive data | 14* | (a) Give characteristics of study participants (eg demographic, clinical, social) and information on exposures and potential confounders | 7 | Demographic characteristics of all subjects are summarized in **Table 1**. A total of 306 subjects, including 156 males (51.0%) and 150 females (49.0%), were eligible for analysis. The mean age was 38.1 ± 9.5 years (range 19-66 years). |
|  |  | (b) Indicate number of participants with missing data for each variable of interest | NA |  |
|  |  | (c) *Cohort study*—Summarise follow-up time (eg, average and total amount) | NA |  |
| Outcome data | 15* | *Cohort study*—Report numbers of outcome events or summary measures over time | *NA* |  |
|  |  | *Case-control study—*Report numbers in each exposure category, or summary measures of exposure | *NA* |  |
|  |  | *Cross-sectional study—*Report numbers of outcome events or summary measures | *8* | **Table 1** presents the average value of L1-L2 vBMD, L1-L2 BMAT and abdominal adipose tissue. vBMD for male group was 147.1±30.4 mg/cm^3^ and for female group was 159.3±34.4 mg/cm^3^. |
| Main results | 16 | (*a*) Give unadjusted estimates and, if applicable, confounder-adjusted estimates and their precision (eg, 95% confidence interval). Make clear which confounders were adjusted for and why they were included | 8 | As shown in **Table 3**, the relationships between different abdominal adipose tissue distribution, BMAT and vBMD were further tested using multiple linear regression analysis, with age and gender and BMI adjusted. The R^2^ and adjusted R^2^ of the overall linear model was 0.545 and 0.532, respectively (*p* < 0.05). |
|  |  | (*b*) Report category boundaries when continuous variables were categorized | NA |  |
|  |  | (*c*) If relevant, consider translating estimates of relative risk into absolute risk for a meaningful time period | NA |  |

Continued on next page

| Other analyses | 17 | Report other analyses done—eg analyses of subgroups and interactions, and sensitivity analyses | 8 | As shown in **Figure.4** and **Table 2**, Pearson’s correlation coefficients between age, BMI, abdominal adipose tissue, PDFF of BMAT were presented. |
| --- | --- | --- | --- | --- |
| Discussion | | | | |
| Key results | 18 | Summarise key results with reference to study objectives | 9 | Our findings revealed that age, BMAT, VAT and TAT were significantly negatively correlated with vBMD. Furthermore, the study identified VAT, SAT and BMAT as independent factors of vBMD. These findings suggest there is a close and interconnected relationship between abdominal adipose tissue, BMAT, and vBMD. |
| Limitations | 19 | Discuss limitations of the study, taking into account sources of potential bias or imprecision. Discuss both direction and magnitude of any potential bias | 12 | The present study has several limitations. |
| Interpretation | 20 | Give a cautious overall interpretation of results considering objectives, limitations, multiplicity of analyses, results from similar studies, and other relevant evidence | 11 | Lastly, our results indicate that abdominal adipose tissue may exert a dual effect on BMD, whereby VAT has a detrimental effect on bone, while SAT are beneficial. In the future prevention and management of osteoporosis, more attention should be paid to the distribution of local adipose tissue rather than just focusing on total body weight or fat mass, with specific monitoring of VAT. Future research should continue to explore these relationships to better understand the mechanisms and implications of abdominal adipose tissue on bone and to develop targeted interventions for high-risk populations. |
| Generalisability | 21 | Discuss the generalisability (external validity) of the study results | 11 | Although osteoporosis is more commonly diagnosed in elderly women, it is crucial to recognize that bone loss is a universal issue that begins earlier in life for both sexes. Understanding the distinct mechanisms and risk factors associated with bone loss in men and women can facilitate the development of effective prevention and treatment strategies tailored to individual needs. |
| Other information | |  | | |
| Funding | 22 | Give the source of funding and the role of the funders for the present study and, if applicable, for the original study on which the present article is based | NA | Relevant funding information was on the website |

*Give information separately for cases and controls in case-control studies and, if applicable, for exposed and unexposed groups in cohort and cross-sectional studies.

**Note:** An Explanation and Elaboration article discusses each checklist item and gives methodological background and published examples of transparent reporting. The STROBE checklist is best used in conjunction with this article (freely available on the Web sites of PLoS Medicine at http://www.plosmedicine.org/, Annals of Internal Medicine at http://www.annals.org/, and Epidemiology at http://www.epidem.com/). Information on the STROBE Initiative is available at www.strobe-statement.org.
